# Perceptual discrepancies in the experience and reporting of violence against children are more pronounced among social workers compared to laypeople

**DOI:** 10.1371/journal.pone.0336900

**Published:** 2025-12-22

**Authors:** Mats Dahl, Leonard Ngaosuvan, Jessika Granberg Flintberg, Johanna Silfversparre, Lotta Stille, Marta Lasota, Sverker Sikström

**Affiliations:** 1 Department of Psychology, Lund University, Lund, Sweden; 2 Department of Culture and Society, Division of Social Work (SOCARB), Linköping University, Linköping, Sweden; 3 Center for Research on Personality Development, SWPS University, Poznań, Poland; NYU Grossman School of Medicine: New York University School of Medicine, UNITED STATES OF AMERICA

## Abstract

When violence against a child is reported to authorities, it is crucial that its severity is communicated effectively and evaluated accurately by professionals. This study, conducted in two phases, investigated perceptual differences in calibration and accuracy regarding psychological and physical violence against children among parents, social workers, and laypeople. In Phase 1, parents who had witnessed violence against their children, committed by a current or former partner, provided first-person narratives describing these events and rated the severity of the violence. In Phase 2, social workers and a control group (laypeople) read these narratives and rated the severity of the violence. Previous research on intimate partner violence suggests that recipients often underestimate the severity of psychological violence and overestimate that of physical violence. We hypothesized that this pattern would also apply for the control group, whereas social workers, due to their professional experience, would show smaller discrepancies compared to the parents. The results partially supported our hypotheses. The control group overestimated the severity of physical violence but did not significantly underestimate that of psychological violence. Unexpectedly, social workers overestimated the severity of psychological violence, resulting in larger perceptual discrepancies than the control group, contrary to the hypothesis. They also overestimated the severity of physical violence, but their ratings did not differ significantly from the control group. These findings suggest that professional experience does not necessarily reduce perceptual biases in assessing violence severity and may, in some cases, amplify them. This study highlights the complexities professionals face in evaluating violence against children and the need for further research, particularly considering potential influences such as desensitization, cognitive biases, and cultural factors.

## Introduction

To better protect children from abusive parents, it is crucial that social workers, legal authorities, and professionals to whom children disclose their experiences accurately assess the severity of the violence [1]. However, the information that child protection social workers evaluate in their daily decision-making is often complex, and cognitive biases can influence their ability to make objective judgments. These biases have been shown to critically affect key decisions, such as those regarding permanency of childcare [2,3], making the development of reliable methods for assessing and addressing both the violence and its underlying causes a significant challenge [4]. In child custody cases, inaccurate evaluations of violence may result in unjustified separations of children from parents or, conversely, children being placed in harmful situations [5]. Moreover, all parties involved in these cases must share a consistent understanding of the severity of the violence. If they do not, assessments and court rulings risk being confusing, inconsistent, undermining the rule of law. The assessment of violence severity therefore has far-reaching societal implications, influencing not only how laws are developed and enforced but also how resources are allocated to social workers, legal jurisdictions, and law enforcement [6]. Given these challenges, it is essential to investigate how physical and psychological violence against children is communicated by parents and perceived by third parties, particularly social workers and other professionals who regularly encounter such information and make decisions related to child abuse.

### Child abuse

Research on violence against children (VAC) reveals complex, region-specific trends influenced by socioeconomic and political factors. A ten-year study in the UK found that violence-related injuries among children have increased in tandem with cuts to youth services funding, suggesting an economic link between budget reductions and heightened violence-related trauma [7]. In the U.S., data from 1997 to 2020 indicates a general decline in reported cases of physical abuse, though this trend has been partly attributed to the implementation of alternative response protocols, which have helped maintain lower levels [8]. In Sweden, violence against children has decreased over the past 40 years, yet it remains a significant concern [4,9]. Collectively, these studies show that while some regions have successfully reduced violence against children, others face increases, often tied to systemic challenges such as insufficient funding and inadequate legal enforcement.

A substantial portion of the physical and psychological violence against children occurs within the home, perpetrated by a parent or legal guardian [10,11]. It is particularly concerning that the most frequent perpetrators are family members, given that children often depend on them and may experience a strong sense of loyalty toward these individuals. [11].

Parental child abuse has profound global consequences for public health and social welfare and constitutes a violation of children’s human rights. Research consistently reveals severe, enduring consequences for children’s mental and physical health, with effects persisting from childhood into adulthood. For instance, children exposed to parental abuse or domestic violence face heightened risks for mental health issues such as low self-esteem, depression, and anxiety, alongside substance abuse, and challenges in social and economic domains, with intergenerational patterns often emerging [12–14]. As part of global efforts to combat the multifaceted impacts of parental child abuse, the United Nations adopted the “Convention on the Rights of the Child” on November 20, 1989. This convention reflects the international community’s commitment to protecting children’s rights and ensuring their well-being [15].

### Types of violence

#### Physical violence.

The World Health Organization (WHO) defines physical violence against children as “the intentional use of physical force against a child that results in – or has a high likelihood of resulting in – harm for the child’s health, survival, development or dignity” [16]. Physical violence is often used as a form of punishment, involving actions such as kicking, pushing, slapping, or beating the child.

The prevalence of physical violence against children is varied and context-specific. A systematic review and meta-analysis estimated that globally, approximately 17.3% of children are victims of physical domestic and family violence, while 16.5% witness such violence by the age of 18. The prevalence was highest in West Asia and Africa, where nearly half of children are affected, and lowest in the Developed Asia Pacific region, with rates as low as 3.7%. Boys were found to be 25% more likely than girls to experience physical violence, though the likelihood of witnessing violence was nearly equal across genders [17]. Sweden offers an example of how legislative and societal changes can influence these trends. As the first country in the world to ban corporal punishment and all forms of violence against children in 1979. Since then reports of child physical abuse have steadily declined, reflecting changing attitudes toward child abuse [4,18]. In the 1960s, nearly every child in Sweden experienced some form of corporal punishment, with over 90 percent of parents resorting to it at least once annually, and a third doing so regularly [19]. By 1980, this figure had dropped to about 50%, and by 2000, only 14% of children were subjected to corporal punishment. However, the decline mainly relates to milder forms of abuse, with more severe cases showing a smaller reduction [4,18,20].

A 2008 population-based study in Sweden on the prevalence of physical violence against children found that 10.8% of surveyed children had witnessed physical violence between adults in their families. Among these children, 58% also reported being physically struck themselves [4]. These findings highlight a strong link between parental intimate partner violence (IPV) and violence against children (VAC), making IPV a significant risk factor for child physical abuse [9]. Research shows that children living in households with IPV are six times more likely to experience physical violence themselves [21].

#### Psychological violence.

Historically, there has been less consensus concerning the definition of psychological violence [22]. Psychological violence can be seen as part of the wider definition of psychological maltreatment (PM). According to the American Professional Society on the Abuse of Children (APSAC), PM is defined as a “repeated pattern or extreme incident(s) of caretaker behavior that thwart the child’s basic psychological development needs (e.g., safety, socialization, emotional and social support, cognitive stimulation, respect) and convey that the child is worthless, defective, damaged, unloved, unwanted, endangered, primarily useful in meeting another’s needs, and/or expendable” [23, p. 4]. A more narrow definition is given by WHO: “Emotional or psychological violence includes restricting a child’s movements, denigration, ridicule, threats and intimidation, discrimination, rejection, and other non-physical forms of hostile treatment” [24]. Moreover, less research has been conducted on psychological violence. This could potentially skew statistics on psychological violence, as reporting often depends on the victim’s awareness of what constitutes violence. Another related issue is that, in several cultural contexts, behaviors that constitute psychological violence are socially accepted as legitimate forms of parenting [22]. Nevertheless, a large body of research supports the conclusion that words can cause just as much harm as physical violence [22,25].

### Evaluation of violence

Research on violence evaluation reveals that ratings are influenced more significantly by the type of violence – whether psychological or physical – committed by the offender than by the victim’s communication of their experience [26,27]. Specifically, psychological violence is typically rated as less severe by individuals to whom it is communicated compared to those who experience it first-hand, while physical violence tends to receive higher severity ratings from recipients of the information than from victims themselves [5,27–31]. One study found that 72% of female victims considered psychological violence more harmful and impactful than physical violence [32]. Consistent findings emerge in studies of young adults exposed to both types of violence in childhood, where psychological abuse was perceived as more detrimental [33]. This discrepancy suggests challenges in effectively communicating the impact of psychological violence, as recipients may struggle to grasp its effects on victims [5,27,29,31].

One explanation for this may lie in the invisibility of psychological violence, which lacks the overt signs typical of physical violence (e.g., bruises, broken bones), thus complicating the understanding of its effects on victims. Furthermore, psychological violence often functions as an indirect reinforcer, being identified as violent only when its impact on the victim is apparent [27]. For instance, a raised voice might appear highly threatening in one setting but inconsequential in another, making psychological violence inherently harder to define compared to physical violence, which is typically a direct reinforcer where harm is immediately recognized. The challenge in recognizing and delineating psychological violence is particularly concerning, as evidence suggests it is not only the most common form of violence against children [9], but also perceived by victims as particularly harmful [32–33].

The perceived severity of violence may also be influenced by the identity of the narrator describing the violent event. For instance, Sikström and Dahl [27] report that correlations between severity ratings by narrators and recipients are lowest for offender narratives, as offenders tend to rate their actions as less severe than victims do. This finding aligns with prior studies indicating that offenders, regardless of gender, may downplay the severity of their actions due to denial and a tendency to shift blame onto the victim [34–36]. This pattern suggests that perpetrators might intentionally provide vague accounts of violent events to evade accountability [34,37–40].

Festinger’s theory of cognitive dissonance [41] supports a similar interpretation, positing that individuals experience psychological discomfort when confronted with conflicting beliefs, prompting efforts to alleviate dissonance. This dissonance reduction can occur through selective avoidance of conflicting information or modification of one’s beliefs for consistency [42]. This framework may account for offenders’ tendencies to rate severity lower and provide vague descriptions, as these behaviors serve to internally rationalize their violent actions [27,35].

Similarly, the parental role introduces unique complexities in evaluating the severity of violence. Research highlights that parents often struggle to process their child’s disclosure of violence or abuse, grappling with intense psychological distress and emotional reactions such as guilt, self-blame, and a sense of failure in their protective role [43]. This internal conflict can influence how they interpret and respond to the abuse. These findings suggest that parents’ emotional turmoil and need to reconcile their identity as protectors can deeply affect their perception of the incident’s severity.

Sikström and Dahl [27] also found that bystanders often attribute higher severity to physical violent acts than victims do. These perceptual differences in calibration, or variance in perceived severity, may also be understood within the framework of cognitive dissonance [44–46]. For example, one literature review [47] suggests that victims of intimate partner violence (IPV) may understate the severity of their partner’s aggression, potentially due to cognitive dissonance arising from their decision to remain in a relationship with an aggressive partner. Cognitive dissonance theory, therefore, would predict calibration differences in narratives from victims and bystanders but not necessarily from offenders, who may minimize severity in their narratives, resulting in no substantial difference between their severity ratings and those of third parties.

Moreover, victims’ tendencies to rate violence as less severe than bystanders align with the theory of desensitization, which posits that repeated exposure to violence can lead to a reduced sensitivity to its severity. Victims, therefore, may rate severity lower due to their own experiences, compared to bystanders who have not been repeatedly exposed to violence. This desensitization suggests that perceptual differences will be manifested in narratives from both victims and offenders, as well as from bystanders who frequently witness violence. As a result, while descriptions of violent events may remain consistent, individuals with greater exposure to violence might assign lower severity ratings due to habituation [27,48–50].

### Hypotheses

The aim of this study is to investigate perceptual differences in severity ratings of violence towards children, as assessed by parents, social workers, and a control group. We investigate differences in calibration and accuracy of severity ratings of psychological and physical violence. Parents provide first-person narratives recounting their experiences of witnessing harm inflicted on their children by a current or former partner. The focus is on how the severity of these narratives is conveyed after being read and rated by social workers, or a control group of general population. Thus, the raters in our study comprise three groups: Parents providing and rating their narrative and two receiving groups: one consisting of professional social workers and the other of laypeople. The term “social workers” will, in this study, refer to individuals with professional experience in addressing issues related to violence against children, whereas the term “laypeople” is used here to denote the control group for comparison with social workers. It is important to note that laypeople, in this context, do not possess the same professional experience or background in addressing violence against children as the social workers. This group will hereafter be referred to simply as the control group. The severity ratings provided by the three different groups (narrators, social workers, and control group) will be compared to assess how perceptual differences in calibration (H1) and accuracy (H2) vary by type of violence.

Discrepancies between severity ratings indicate perceptual differences, which can have negative implications for both parents and children in violent situations seeking professional help or support. Overestimating the level of violence could lead to legal or interpersonal consequences for the perpetrator, while underestimating it might endanger the child by disregarding the risks they face. Accurate communication of the severity of violence against children is essential for the appropriate evaluation of offenders and victims in contexts such as court trials and, specifically, custody cases. This study will therefore investigate the following hypotheses:

#### H1. Perceptual differences in calibration.

a Psychological violence. The control group and the social workers are expected to underestimate the severity of psychological violence compared to the narrators, resulting in a negative perceptual differences score (PDC).
b Social workers compared to the control group (psychological violence). Due to their experience, social workers are hypothesized to exhibit smaller perceptual differences scores (PDC) for psychological violence compared to the control group.
c Physical violence. The control group and the social workers are expected to overestimate the severity of physical violence compared to the narrators, resulting in a positive perceptual differences score (PDC).
d Social workers compared to the control group (physical violence). Due to their experience, social workers are hypothesized to exhibit smaller perceptual differences scores (PDC) for physical violence compared to the control group.

#### H2. Perceptual differences in accuracy.

a Type of violence. Severity ratings are expected to be evaluated less accurately for psychological violence than for physical violence. The correlation between experience and communicated severity ratings is hypothesized to be lower for psychological violence than for physical violence, indicating that psychological violence is harder to evaluate and hence more affected by perceptual differences in accuracy (PDA).
b Social workers compared to the control group (psychological violence). Due to their experience, social workers are hypothesized to demonstrate smaller perceptual differences in accuracy (PDA) for psychological violence compared to the control group – reflected in higher correlations between experienced and communicated severity ratings.
c Social workers compared to the control group (physical violence). Similarly, social workers are hypothezised to exhibit smaller perceptual differences in accuracy (PDA) for physical violence compared to the control group.

## Method

### Design

The study employed a mixed design, with witnessed violence (narrators) versus communicated violence (readers) as the between-group variable, and type of violence (psychological and physical) as the within-group variable. The dependent variable was the severity rating of the violence. The study was conducted in two phases: Phase 1, where narratives were collected, and Phase 2, where the narratives were read and rated. The ratings were not shared among respondents.

### Participants

#### Phase 1.

For Phase 1 we used data collected by Lasota et al. [51]. Participants were pre-screened using Prolific Academics (www.prolific.com) based on their first language (English), location (UK or USA), and relationship status (married, divorced, or separated). An additional criterion required that only adults with children aged 18 years or younger were eligible. Participants recruited for Phase 1 were compensated £4.50. Among the 167 narrators, 88 were women (55.3%), 69 were men (43.4%), three identified as “Other” (1.3%), and there were eight missing values. The mean age was 39.6 years. For a more detailed description, see Lasota et al. [51].

#### Phase 2.

Participants in the control group were also recruited from Prolific Academics (www.prolific.com) and were pre-screened with the following criteria: age (18–100 years), language (fluent in Swedish), and current location (Sweden). Upon completion, participants in the control group were compensated £1.34 (£8.04 per hour) for their participation. Among the 285 participants in the control group, 85 were women (30.1%), 195 were men (68.8%), three identified as “Other” (1.1%), and there were two missing values. The mean age was 31.5 years.

The social workers had the same inclusion criteria as the control group. However, an additional inclusion criterion required that participants should be actively working or have previously worked in an area involving stories of violence against children. Upon completion, the social workers were given the option of receiving a cinema ticket as compensation. Of the 96 social workers, 70 were women (77.8%), 18 were men (20%), two identified as “Other” (2.2%), and there were seven missing values. The mean age was 39.2 years. Sixteen different job titles were identified within this group, with “social secretary,” “family law secretary,” and “lawyer” being the most common titles, in that order.

All participants were informed about the purpose of the study, data confidentiality, and data storage before their participation. They then signed a consent form to participate in the study.

### Material and procedure

Phase 1 narratives depicting instances of psychological and physical violence against children were taken from Lasota et al. [51], in which narrators were parents detailing four forms of violence: psychological and physical violence they had committed (offenders), as well as psychological and physical violence their partners had perpetrated against the child (bystanders). Narrators were instructed to write text paragraphs describing events of psychological and physical violence against children and to rate the severity of the violence from 0 (not severe at all) to 10 (very severe). In the current study, only narratives describing violence perpetrated by the narrator’s current or ex-partner were included (bystander role). This decision was made due to its relevance to scenarios social workers are most likely to encounter and because the majority of narratives fell into this category.

The narratives were reviewed, and those not meeting the bystander-role criterion were excluded. Narratives in which narrators misclassified physical violence as psychological, or vice versa, were reclassified and moved to the correct category. In total, 253 narratives were included in our surveys, with 138 concerning psychological violence and 115 concerning physical violence. Since these narratives were originally written in English, they were translated into Swedish using the Deepl tool (www.deepl.com) and subsequently reviewed and manually edited by the authors.

In Phase 2, two additional groups of participants – the social workers (N = 87) and the control group (N = 280) – read and rated the narratives collected in Phase 1. Two versions of a survey were created on Qualtrics (www.qualtrics.com): one for the social workers and one for the control group. The surveys were similar, but slightly modified, from previous studies by our research group to better suit the current purpose, allowing for a fair comparison, reliability and validity to earlier publications.

Two pilot studies were conducted before finalizing the surveys. The data from the first pilot study were excluded from data analysis due to survey changes made based on respondent feedback. Data from the second pilot study were included in the control group, as no further changes to the survey were necessary.

The social worker survey was distributed to social workers via an email list. Additionally, the survey was promoted on LinkedIn (www.linkedin.com) to individuals meeting our criteria (listing their occupation as social worker), who were contacted and informed about the study. The control group survey was distributed via Prolific Academics (www.prolific.com).

Both surveys opened with an introduction explaining the aim of the study and providing information on anonymity and informed consent. The narratives from Phase 1 were randomized and evenly distributed within each survey. Participants were instructed to read one narrative each depicting psychological violence and one depicting physical violence, then rate the severity on a scale from 0 (not severe at all) to 10 (very severe). Lastly, participants provided demographic information, such as gender, age, number of children, level of education, occupation, and citizenship.

At the end of the survey, social workers were directed to a separate reimbursement survey, allowing them to provide their email address to receive a cinema ticket if they wished. The reimbursement survey was created to ensure participant anonymity; by using a separate, anonymous survey link, email addresses could not be traced back to the responses in the social worker survey.

At the end of the control group survey, participants were asked to provide their Prolific ID and the specific survey code to receive their payment.

### Data analysis

After reviewing the responses from Phase 2, 35 responses from the control group and 44 from the social worker group were excluded due to missing ratings and/or failure to meet the inclusion criteria. The final number of participants included in the study was 87 in the social worker group and 280 in the control group.

The severity ratings provided by narrators were paired with recipient ratings for the same narratives. As there were two groups in Phase 2, this process resulted in three rating variables per narrative (i.e., parents from Phase 1, and either social workers or control group from Phase 2). Since some narratives received more than one rating in Phase 2, the number of ratings included in the recipient ratings variable was correspondingly higher. Therefore, Phase 1 ratings were duplicated as needed and matched with their Phase 2 counterparts.

## Results

### H1. Perceptual differences in calibration

Perceptual differences in calibration (PDC) measures the paired difference between the severity rating of the narrator in Phase 1 and the rater in Phase 2. To examine perceptual differences in calibration (H1), we began by comparing the psychological and physical violence ratings across the three groups (see Table 1).

**Table 1:** Severity ratings of violence for narrators and recipients.

Type of violence	Narrators	Control group	Social workers
	Mean (SD)(N)	Mean (SD)(N)	Mean (SD)(N)
Psychological violence	4.95 (2.99)	4.86 (2.74)	5.70 (2.55)
Physical violence	4.83 (3.22)	6.44 (2.73)	6.78 (2.69)
Psychological violence women	5.34 (3.12)(156)	5.40 (2.73)(85)	5.84 (2.57)(70)
Psychological violence men	4.43 (2.75)(115)	4.63 (2.57)(195)	5.17 (2.46)(18)
Physical violence women	5.42 (3.19)(157)	6.78 (2.66)(85)	6.79 (2.74)(70)
Physical violence men	4.02 (3.09)(114)	6.30(2.75)(195)	6.78 (2.56)(18)

The table displays the types of violence, mean (Mean), standard deviation (SD) and the number of matching pairs (N) for violence severity ratings for narrators, control group, and social workers. The first two rows summarize data for all participants, while subsequent rows break down the ratings by gender of the respondents.

**Fig 1:**
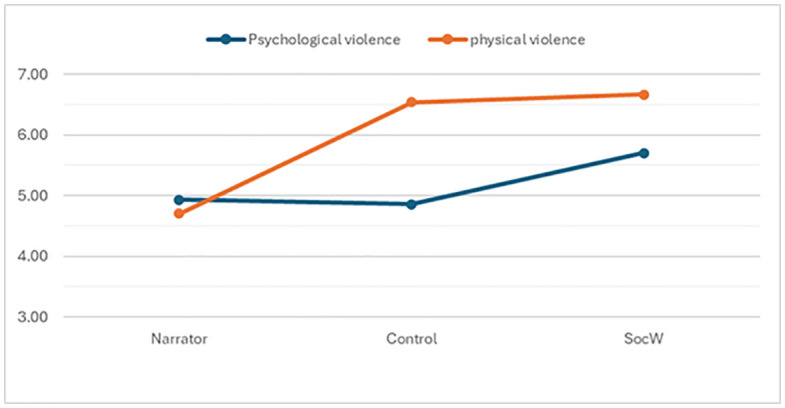
Severity ratings of violence for narrators and recipients. The figure shows the severity ratings of psychological (blue) and physical (orange) violence as assessed by narrators, the control group, and social workers.

Hypotheses H1a and b posited that both social workers and the control group would underestimate the severity of psychological violence compared to the narrators, and that social workers would exhibit smaller perceptual difference scores (PDC) for psychological violence compared to the control group. Social workers generally provided the highest severity ratings (M = 5.70, SD = 2.55), followed by the the narrators (M = 4.93, SD = 2.99), and finally, slightly below, the control group (M = 4.86, SD = 2.74)(see Fig 1). This indicates that the social workers rated the severity higher than the narrators and the control group, contradicting the hypothesis that they would underestimate psychological violence. A one-way ANOVA revealed a significant difference between the three groups (F(2,276) = 4.13, p = .017). Post-hoc analyses (Games-Howell corrected for unequal variance) showed that social workers rated psychological violence as significantly more severe than narrators (p = .013), which is contrary to H1b. No significant differences were found between the control group and the narrators, nor between the control group and social workers. A perceptual difference in calibration was therefore identified, but not in the direction hypothesized in H1b. Thus, both H1a and H1b were rejected, as no support was found.

Hypotheses H1c and d suggested that both social workers and the control group would overestimate the severity of physical violence compared to the narrators, and that social workers would exhibit smaller perceptual difference scores (PDC) for physical violence compared to the control group. Social workers generally provided the highest ratings (M = 6.60, SD = 2.72), followed by the control group (M = 6.43, SD = 2.74), and lastly the narrators (M = 4.88, SD = 3.22)(see Fig 1). A one-way ANOVA showed a significant difference between the three groups (F(2,254) = 25.1, p < .001). Post-hoc analyses (Games-Howell corrected for unequal variance) showed that both social workers and the control significantly overestimated the physical violence (narrators vs. control group p < .001; narrators vs social workers, p < .001). No difference was found between the control group and the social workers. Therefore, H1c was supported. However, contrary to H1d, social workers showed greater perceptual differences in calibration for physical violence than the control group, as the difference between their ratings and the narrators’ ratings was larger. Nevertheless, the greater calibration difference observed for social workers contradicts the expectation that the control group would show more perceptual differences in calibration than the social workers. Thus, while H1c was supported, H1d was not, as the results indicate that social workers showed greater perceptual differences than the control group.

### Gender differences

A two-way ANOVA (Gender (man, woman))*(Group (narrator, control, social worker)) of the rating of psychological violence showed no interaction effect, but a main effect of gender, F (1,633) = 6.74, p = .01; η² = .01). No main effect of group was found. However, the follow-up post hoc analyses showed no significant differences when comparing the three different groups separately. A corresponding ANOVA analysis (Gender (man, woman)) *(Group (narrator, control, social worker)) of the ratings of physical violence showed no interaction effect, but a main effect of gender, F (1,633) = 4.14, p = .04; η² = .006) and a main effect of group, F (1,633) = 28.77, p < .01; η² = .082). The following contrast analysis showed that violence was rated higher by social workers than by both the control group and the narrators (p < .01). Also, when comparing the narrator group the post-hoc analysis revealed that women rated physical violence significantly higher than men (p < .01), something that was not true in the control group or the group of social workers (see Table 1).

**Fig 2:**
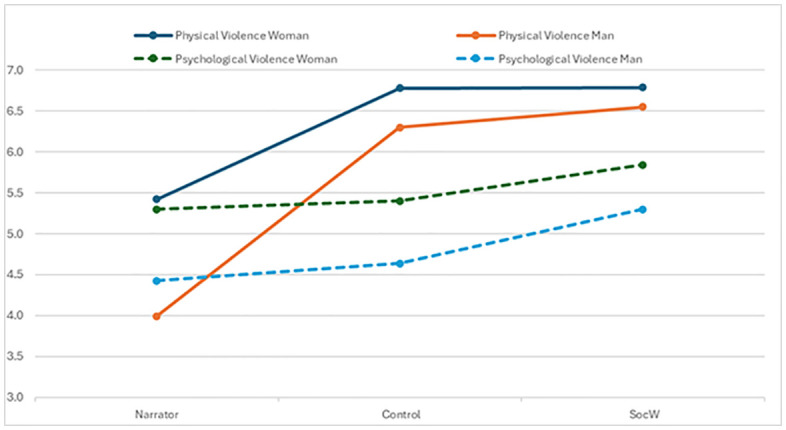
Severity ratings of violence by gender for narrators and recipients.

Fig 2 (see Fig 2) shows the severity ratings of psychological (dashed lines) and physical (solid lines) violence, broken down by gender, as assessed by narrators, the control group, and social workers.

### H2. Perceptual difference in accuracy

Perceptual difference in accuracy (PDA) refers to the discrepancy between the narrator’s severity rating of a violent event and the recipient’s ability to accurately predict that rating. In other words, PDA captures the mismatch in severity ratings between narrators and recipients of the same event, highlighting the difficulty of effectively communicating the severity of violent events. In this study, PDA was measured using Pearson’s correlations between the narrator’s severity rating and the mean severity ratings given by the social workers and the control group, respectively, for the same violent event. These coefficients are presented in a single matrix (see Table 2), where the lower triangle represents correlations for psychological violence and the upper triangle represents correlations for physical violence.

**Table 2:** Pearson’s correlation coefficients for severity ratings of psychological and physical violence among narrators, the control group, and social workers.

Variable	Narrators	Control group	Social workers
Narrators	–	0.416**	0.413**
Control group	0.311**	–	0.401**
Social workers	0.347**	0.434**	–

Pearson’s correlation coefficients. Lower triangle = psychological violence; upper triangle = physical violence.

**p < .001.

Hypothesis H2a posited that perceptual differences in accuracy (PDA) would be greater for psychological violence than for physical violence. Specifically, it was expected that severity ratings would be evaluated less accurately for psychological violence, as indicated by a lower correlation between narrators’ severity ratings and those given by social workers and the control group. The results supported this hypothesis. The correlation between narrators’ ratings and the ratings provided by both social workers and the control group was significantly weaker for psychological violence compared to physical violence, indicating a larger perceptual difference in accuracy. Consequently, these results confirm that psychological violence is more susceptible to perceptual differences in accuracy, supporting H2a (see Table 2).

Hypothesis H2b suggested that social workers, due to their experience, would demonstrate smaller perceptual differences in accuracy (PDA) for psychological violence compared to the control group. This would be reflected in a higher correlation between social workers’ severity ratings and those of the narrators, compared to the correlation between the control group and the narrators. The results did not support this hypothesis. The correlation between social workers’ and narrators’ ratings for psychological violence was weak (r = 0.347, n = 286, p < .001), as was the correlation between the control group and narrators (r = 0.311, n = 286, p < .001). The difference between these two correlations was not statistically significant. Consequently, H2b was rejected.

Hypothesis H2c posited that social workers would demonstrate smaller perceptual differences in accuracy (PDA) for physical violence compared to the control group. This would be reflected in a higher correlation between social workers’ severity ratings and those of the narrators, compared to the correlation between the control group and the narrators. The results did not support this hypothesis. A moderate correlation was found between social workers’ and narrators’ ratings for physical violence (r = 0.413, n = 286, p < .001), which was nearly identical to the correlation between the control group and narrators (r = 0.416, n = 286, p < .001). The difference between these two correlations was not statistically significant. Consequently, H2c was rejected.

## Discussion

The aim of this study was to investigate perceptual differences in how the severity of violence against children, both psychological and physical, is evaluated by narrators (parents), social workers, and a control group. Specifically, this research focused on differences in calibration and accuracy of severity ratings and sought to explore how these differences may impact the communication and understanding of such violence, particularly in professional settings involving child welfare.

### H1. Perceptual differences in calibration

**H1a** hypothesized that both social workers and the control group would underestimate the severity of psychological violence compared to narrators. For the control group, the results showed that this difference was not significant, and warrants further investigation. Despite the lack of statistical significance, the finding that the control group rated psychological violence the lowest aligns with previous research suggesting that recipients tend to assign lower severity ratings for psychological violence than narrators [5,27–31]. One possible explanation for this is that psychological violence is not yet as recognized by the public compared to physical violence [52]. Furthermore, psychological violence can function as an indirect reinforcer and is challenging to define because its classification as violence often depends on its consequences, which vary based on situational context [27]. Additionally, prior research suggests that psychological violence is harder to communicate and understand compared to physical violence [5,29,31]. It often requires greater contextual knowledge about the relationship between the offender and the victim, as well as the broader social context, to fully grasp its scope [27].

While the control group did not significantly differ in their ratings of the severity of psychological violence, the social workers displayed an unexpected pattern: a ‘reversed’ perceptual difference in calibration. Contrary to the hypothesis, social workers rated psychological violence as the most severe of all groups, and the difference in severity ratings between social workers and narrators was statistically significant. This is an intriguing finding that needs to be corroborated in future independent studies. Several possible explanations could account for this pattern of results. Social workers are likely more informed and educated about the definitions and consequences of psychological violence, and more experienced readers or listeners of such narratives. This professional knowledge and experience may contribute to their higher severity ratings compared to narrators and laypeople. Additionally, social workers are trained to address violence in all forms, which may foster greater vigilance and sensitivity toward recognizing and evaluating psychological violence. This heightened awareness, coupled with a deeper understanding of what constitutes psychological violence and its detrimental effects, could explain their higher severity ratings.

For **H1b**, which hypothesized that social workers would show smaller perceptual differences in calibration compared to the control group, the results indicated the opposite. Social workers exhibited greater perceptual differences in calibration compared to narrators than the control group did. As a result, H1b was rejected. This unexpected finding is interesting and may, in part, be explained by the desensitization of narrators. If narrators are desensitized due to repeated exposure to violence, their ratings may reflect an underestimation of its severity [48–50]. Consequently, social workers’ higher ratings might not necessarily indicate overestimation but rather a closer alignment with the actual severity of the violence. Future research could investigate this further by comparing social workers’ ratings to an objective measure of violence severity, potentially using AI, to assess whether their evaluations are more aligned with objective assessments than those of narrators. Such research could also help corroborate the hypothesis that narrators’ ratings are influenced by desensitization.

**H1c** hypothesized that both social workers and the control group would overestimate the severity of physical violence in comparison to narrators. The results supported this perceptual difference in calibration (PDC), as hypothesized. This finding is consistent with previous research indicating that recipients tend to rate physical violence as more severe than narrators do [5,27–31]. One possible explanation for why recipients rate physical violence higher is that physical acts are more readily identified as violence, given its nature as direct reinforcers. Additionally, physical violence often leaves visible marks, such as bruises, broken bones, or wounds, which makes the negative consequences for the victim more tangible and easier to comprehend [27].

However, contrary to **H1d**, which hypothesized that social workers would show smaller perceptual differences in calibration compared to the control group, the results indicated the opposite. Social workers exhibited greater perceptual differences in calibration for physical violence than the control group, as the difference between their ratings and those of the narrators was larger. This finding suggests that professional experience as a social worker does not necessarily attenuate perceptual differences in calibration. Instead, it may amplify them, possibly due to heightened sensitivity to violence based on professional training and exposure.

This pattern of results, particularly the findings for H1a and H1c, aligns with the prediction made by the theory of desensitization: exposure to violence lowers the perceived severity of violent events [48–50]. The results indicate that narrators do not minimize the severity of the violence in their written narrative, as evidenced by the fact that social workers rated the violence as more severe. However, narrators may fail to recognize the true severity of the events when providing their rating due to desensitization from frequent exposure to violence [50]. The narrator’s desensitization, reflected in their tendency to provide lower severity ratings, may also be linked to the strong association between violence against children and intimate partner violence (IPV) within the parental relationship. This association suggests that both adults and children are at heightened risk when violence occurs within the household. Many parents in the sample may themselves be victims of violence, which could further amplify the effects of desensitization [4,9].

The results for physical violence demonstrate a stronger tendency for desensitization compared to psychological violence, as narrators provided the lowest ratings for physical violence among all groups. However, the less pronounced desensitization effect for psychological violence is not surprising, given that psychological violence is more challenging for recipients to comprehend [5,27,29,31].

Another interesting finding, consistent with previous research [5,27–31], is that narrators generally rated psychological violence (M = 4.95) as more severe than physical violence (M = 4.83). This aligns with earlier studies indicating that both women [32] and children [33] who have experienced violence often perceive psychological violence as worse and more harmful than physical violence. These findings are consistent with our data, suggesting that psychological violence is perceived as more severe by narrators. However, in this study, narrators are not assessing the severity of violence directed at themselves, but at their children. It is important to note that parents who witness violence against their child may also be victims themselves, as there is significant overlap between violence against children (VAC) and intimate partner violence (IPV). This overlap could influence their perception, leading them to rate psychological violence against their child as more severe than physical violence. This finding emphasizes the need for heightened awareness and increased knowledge regarding psychological violence against children and how it is communicated.

### H2. Perceptual differences in accuracy

**H2a** hypothesized that perceptual differences in accuracy would be more pronounced for psychological violence than for physical violence. This hypothesis was supported, as psychological violence showed the lowest perceptual difference in accuracy, reflected in the weakest correlation between narrators’ and recipients’ severity ratings. As suggested earlier, one possible explanation for this finding is the inherent difficulty in effectively communicating the impact of psychological violence, as well as the challenges recipients face in fully understanding its effects [5,27,29,31].

Although all correlations were significantly greater than zero, indicating some predictive ability of recipients’ ratings based on narrators’ ratings, the correlations were generally low to moderate. This indicates that the predictive accuracy remains limited. The results suggest that it is challenging to make precise evaluations of severity based solely on written narratives, particularly for psychological violence, which may lack the concrete markers often associated with physical violence.

**H2b** proposed that perceptual differences in accuracy would be smaller between social workers and narrators than between the control group and narrators, as reflected in a stronger correlation between social workers’ and narrators’ severity scores for psychological violence. However, the findings were not significant, and H2b was therefore rejected. The observed difference in correlation strength between the two groups was relatively small, with both correlations being weak. This result suggests, consistent with our other findings, that accurately predicting the severity of psychological violence from narratives remains a challenge, regardless of professional experience or familiarity with cases involving violence towards children.

**H2c** proposed that social workers would show less perceptual differences in accuracy than the control group, meaning that the correlation between social workers’ and narrators’ severity scores for physical violence would be stronger than the corresponding correlation for the control group and narrators. However, the findings were not significant, and this hypothesis was therefore not supported. In contrast, the correlation between the control group and narrators was stronger, suggesting that the control group demonstrated less perceptual differences in accuracy and their ratings were more aligned with the narrators. Social workers, on the other hand, rated the severity of physical violence higher than narrators. One possible explanation for this finding is that social workers, due to their professional experience and understanding of violent situations and their consequences, may be more attuned to the broader impact of physical violence, leading to higher severity ratings.

These results further support the reasoning discussed earlier in relation to H1a and H1c, where narrators appear to provide the lowest severity rating for physical violence, potentially due to desensitization from repeated exposure to violence. This desensitization may lead narrators to underestimate the severity of physical violence compared to recipients, particularly social workers.

### The role of gender in severity ratings

Beyond the primary hypotheses, we also examined the potential influence of gender on severity ratings, as gender distribution varied across groups. Gender composition across groups may have influenced the results, as the proportion of women varied: 78% in the social worker group, 30% in the control group, and 55% in the narrator group. Importantly, the interaction analyses did not reveal any significant interaction effects between gender and group for either psychological or physical violence.

For psychological violence, a very small main effect of gender was found (η² = .01), indicating that, overall, women rated psychological violence as slightly more severe than men. However, post hoc analyses did not reveal any significant gender differences within individual groups (narrators, control group, or social workers). This suggests that while gender had a general influence on severity ratings, the effect was very limited and unlikely to have a meaningful impact on how psychological violence was perceived.

For physical violence, a negligible main effect of gender was found (η² = .006), where women rated physical violence as slightly more severe than men. However, this effect was only significant within the narrator group: post hoc analyses showed that female narrators rated physical violence as significantly more severe than male narrators. No significant gender differences were found within the control group or among social workers, suggesting that gender-based differences in severity perception primarily emerged among those who had personally witnessed the violence. Additionally, a small main effect of group was found for physical violence (η² = .082), where social workers rated physical violence as significantly more severe than both the control group and the narrators. This suggests that professional experience influenced severity ratings more strongly than gender, at least in the context of physical violence.

Given these findings, it appears that gender had a limited impact on severity ratings, whereas group membership (i.e., being a social worker, a layperson, or a witness to violence) played a more substantial role – particularly in the assessment of physical violence. The absence of significant gender differences within the social worker group suggests that their higher severity ratings were likely more influenced by professional experience than by gender alone.

At the same time, women were overrepresented in the social worker group (78%), which aligns with the actual gender distribution in the profession in Sweden [53]. Since social work is a female-dominated field, the gender composition of our sample reflects real-world conditions rather than being an artifact of study design. This means that while gender may have played a minor role in the higher severity ratings observed among social workers, these findings likely reflect patterns that exist in professional settings instead of being a result of sampling bias. Rather than viewing gender and group membership as competing explanations, these results suggest that they are interrelated. While gender may contribute to how individuals perceive and evaluate violence, its effects appear to be overshadowed by professional experience – at least in this context.

### Implications

This study has primarily focused on identifying perceptual differences in severity ratings and contributes to the research area exploring how violence is perceived and evaluated. To our knowledge, no previous studies have directly compared measures of severity for psychological and physical violence against children, highlighting the importance of this study’s findings and its contribution to the field.

Understanding perceptual differences in how violence against children (VAC) is communicated has significant implications across social, cultural, and legal domains. Insights from this research can inform the development and implementations of laws and policies, as well as the allocation of resources to key institutions such as courts, social workers, and law enforcement. Accurate assessments of violence severity are critical to ensuring that victims receive the appropriate support and resources they need. Additionally, such assessments are crucial in determining the correct penalties for perpetrators, ensuring justice and accountability. The evaluation of violence also carries profound implications in custody cases, where decisions regarding the permanency of a child’s living situation or potential separations from a parent may hinge on the accurate communication and understanding of violence against children.

### Limitations

Even though this study provides valuable insights into the perceptual differences in evaluating violence severity among parents, social workers, and laypersons, several limitations must be acknowledged. First, the narratives provided by parents may reflect subjective biases influenced by personal experiences, emotional states, or social desirability, potentially impacting the accuracy of their severity ratings. This subjectivity may be compounded by the lack of an objective benchmark to assess the actual severity of the violent events described.

Second, the study relied on written narratives and ratings, which were assessed in a research setting. Reading and rating narratives under such conditions is not equivalent to real-world decision-making by social workers, where context, follow-up questions, and nonverbal cues are often available. As a result, the data may not fully capture the complexities of professional assessment processes. Psychological violence, in particular, may therefore be underrepresented or misunderstood.

Furthermore, the group of laypersons included in the study may not fully represent the general population in terms of demographic, cognitive, or empathetic profiles. Their interpretations and ratings of violence could therefore vary, which may limit the generalizability of the findings. In addition, while the inclusion of social workers provided valuable insights into professional perceptions, the varying levels of training, experience, and familiarity with violence-related cases within this group could have influenced the consistency of their ratings. The sample size of social workers was also relatively smaller compared to the control group, which may have affected the statistical power of the comparisons. Similarly, the overall sample of parents and professionals was limited in size and demographic diversity (e.g., race, socioeconomic background), which constrains the extent to which the findings can be generalized to broader populations. Finally, differences in cultural norms and legal definitions of violence against children across countries and welfare systems further limit the applicability of the results beyond the specific context studied.

## Conclusion and future research

This study investigated perceptual differences in evaluating the severity of psychological and physical violence against children among social workers, narrators and a control group. The findings reveal several complexities in how different groups assess and understand various forms of violence.

The hypothesis that social workers and the control group (recipients) would overestimate the severity of physical violence compared to narrators was supported. This aligns with previous research showing that recipients tend to perceive physical violence as more severe than those directly experiencing it. Further, our findings suggest that social workers perceive and rate psychological violence as more severe than both the control group and narrators. This tendency may stem from their education and experience in recognizing different forms of violence compared to laypeople.

Interestingly, social workers demonstrated the greatest perceptual differences in calibration and accuracy in our study. This highlights the potential value of future research comparing severity ratings from social workers and narrators against an objective rating tool, such as AI. Such an approach could provide deeper insights into why professionals working with children showed more biases when assessing severity.

The weak and moderate correlations between narrators’ and recipients’ severity ratings further indicate that written descriptions alone may not fully capture the complexity of violence, particularly psychological violence. Future research should investigate perceptual differences in the communication of violence against children using narratives where the narrator is the perpetrator. Such research could have significant implications for legal proceedings, including trials and custody cases, where the accurate assessment of violence severity is critical.

Additionally, future research could further explore how gender and professional training interact in shaping assessments of violence severity, particularly in professions where exposure to cases of child abuse is frequent. While our findings suggest that professional experience had a stronger influence than gender on severity ratings, it remains an open question how these factors interact in different professional contexts. Examining this interplay across various professions, levels of experience, and cultural settings could offer deeper insights into how professionals evaluate violence severity and what factors contribute to potential biases.

## Supporting information

S1 File Data file. (XLSX)
